# Methodological Quality and Reporting of Generalized Linear Mixed Models in Clinical Medicine (2000–2012): A Systematic Review

**DOI:** 10.1371/journal.pone.0112653

**Published:** 2014-11-18

**Authors:** Martí Casals, Montserrat Girabent-Farrés, Josep L. Carrasco

**Affiliations:** 1 CIBER de Epidemiología y Salud Pública (CIBERESP), Barcelona, Spain; 2 Bioestadística, Departament de Salut Pública, Universitat de Barcelona, Barcelona, Spain; 3 Departament de Ciencies Basiques, Universitat Internacional de Catalunya, Barcelona, Spain; 4 Servei d’Epidemiologia, Agència de Salut Pública de Barcelona, Barcelona, Spain; 5 Departament de Fisioteràpia (unitat de Bioestadística), Universitat Internacional de Catalunya, Barcelona, Spain; FIOCRUZ, Brazil

## Abstract

**Background:**

Modeling count and binary data collected in hierarchical designs have increased the use of *Generalized Linear Mixed Models (GLMMs)* in medicine. This article presents a systematic review of the application and quality of results and information reported from GLMMs in the field of clinical medicine.

**Methods:**

A search using the Web of Science database was performed for published original articles in medical journals from 2000 to 2012. The search strategy included the topic “*generalized linear mixed models*”,*“hierarchical generalized linear models”, “multilevel generalized linear model”* and as a research domain we refined by science technology. Papers reporting methodological considerations without application, and those that were not involved in clinical medicine or written in English were excluded.

**Results:**

A total of 443 articles were detected, with an increase over time in the number of articles. In total, 108 articles fit the inclusion criteria. Of these, 54.6% were declared to be longitudinal studies, whereas 58.3% and 26.9% were defined as repeated measurements and multilevel design, respectively. Twenty-two articles belonged to environmental and occupational public health, 10 articles to clinical neurology, 8 to oncology, and 7 to infectious diseases and pediatrics. The distribution of the response variable was reported in 88% of the articles, predominantly Binomial (n = 64) or Poisson (n = 22). Most of the useful information about GLMMs was not reported in most cases. Variance estimates of random effects were described in only 8 articles (9.2%). The model validation, the method of covariate selection and the method of goodness of fit were only reported in 8.0%, 36.8% and 14.9% of the articles, respectively.

**Conclusions:**

During recent years, the use of GLMMs in medical literature has increased to take into account the correlation of data when modeling qualitative data or counts. According to the current recommendations, the quality of reporting has room for improvement regarding the characteristics of the analysis, estimation method, validation, and selection of the model.

## Introduction

Statistical modeling is a highly important tool that receives a lot of attention in any scientific field. In health sciences, statistical models arise as an important methodology to predict outcomes and assess association between outcomes and risk factors as well. Thus, one important aspect is to efficiently test the investigational hypothesis by avoiding biases and accounting for all the sources of variability present in data. This usually leads to complex designs where data is hierarchically structured. Multilevel, longitudinal or cluster designs are examples of such structure. In health sciences, longitudinal studies probably are more common, where measurements are grouped in subjects who are followed over time. Furthermore, other possibilities are studies where measurements are hierarchically grouped in subgroups such as schools, hospitals, neighborhoods, families, geographical areas or place of employment.

In the classic linear model (linear regression analysis, ANOVA, ANCOVA), the variable response is continuous and it is assumed that the response conditioned to covariates follows a normal distribution with maximum likelihood based approaches as the principal estimation methods [1]–[3]. However, the general linear model is not appropriate for non-continuous responses (e.g. binary, counts) because the underlying assumptions of the model do not hold.

Generalized linear models (GLMs) arose as an extension of the classic linear model that allowed for the accommodation of non-normal responses as well as a non-linear relationship between the expectation of the response and the covariates [2], [4], [5]. GLMs are most often applied to count or binary responses in health sciences [6], assuming Poisson, Binomial or Bernoulli as probability distributions for the response.

Similar to the classic linear model (which is indeed a particular type of GLM), GLMs also assume that the observations (conditioned to covariates) are independent and identically distributed. Regarding study designs with hierarchical structure, the assumption of independence is usually violated because measurements within the same cluster are correlated. The main disadvantage of ignoring within-cluster correlation is the bias in point estimates and standard errors. These biases might cause a loss of statistical power and efficiency of hypothesis testing on fixed effects [7], [8]. Thus, the statistical significance could be wrongly assessed [9] and the type I error rate could be different than that *a priori* determined in hypothesis testing.

Generalized linear mixed models (GLMMs) are a methodology based on GLMs that permit data analysis with hierarchical GLMs structure through the inclusion of random effects in the model. The GLMMs are also known in the literature as hierarchical generalized linear models (HGLMs) and multilevel generalized linear models (MGLMs) depending on the field [10]–[12]. For the sake of simplicity we will use the term GLMMs throughout the text. The first estimation method of GLMMs was introduced in the early 1990 s [13]. Nowadays various estimation methods can be found for GLMMs, such as the penalized quasi-likelihood method (PQL) [14], the Laplace method [14], Gauss-Hermite quadrature [15], hierarchical-likelihood methods [11], and Bayesian methods based on the Markov chain Monte Carlo technique [16], [17], and, recently also based on the integrated nested Laplace approximation [18].

Furthermore, GLMM methodology is now available in the main statistical packages, though estimation methods as well as statistical packages are still under development [19], [20].

The increasing interest in GLMMs is reflected by the publication of tutorials in various fields, such as ecology [19], psychology [21], biology [22], and medicine [23]–[26]. Nowadays, original articles, academic work and reports which utilize GLMMs exist, and methodological guidelines and revisions are also available for the analysis of GLMMs in each field [19], [27]–[29].

However, it is not possible to find guidelines that specifically address the appropriate reporting of population modeling studies [30]. In addition, no reviews of the use and quality of reported information by GLMMs exist despite an important increase in quantitative analyses in the academic and professional science settings.

Reporting guidelines are evidence-based tools that employ expert consensus to help authors to report their research such that readers can both critically appraise and interpret study findings [30]–[34]. Recently, minimal rules that can serve as standardized guidelines should be established to improve the quality of information and presentation of data in medical scientific articles [35]. Only Thiele [22] has made reference to GLMMs in the field of biology and still no standardized guidelines indicate what information is relevant to present in medical articles.

For this reason, the objective of the present study is to review the application of GLMMs and to evaluate the quality of reported information in original articles in the field of clinical medicine during a 13-year period (2000–2012), while analyzing the evolution over time, journals, and areas of publication.

## Methods

This review was conducted according to the Preferred Reporting Items for Systematic Reviews and Metanalyses (PRISMA) Statement [36], [37]. We also report the review in accordance with PRISMA guidelines (Checklist S1).

With the objective to obtain and analyze the existing scientific literature related to the use of GLMMs in clinical medicine, a strategic search of original published articles in this field from 2000 to 2012 was performed using the Web of Science database.

The search strategy included the topic “*generalized linear mixed models”*, *“hierarchical generalized linear models”, “multilevel generalized linear model”* and as a research domain we refined by science technology (Appendix S1).

The following fields of clinical medicine were included in the search:

*Endocrinology Metabolism, Urology Nephrology, Public environmental occupational health, Orthopedics, Respiratory system, Entomology, Health care sciences services, Medical laboratory technology, Pediatrics, Pathology, Life sciences biomedicine other topics, Hematology, Geriatrics gerontology, Gastroenterology hepatology, Rheumatology, Critical care medicine, Medical informatics, Emergency medicine, Integrative complementary medicine, Obstetrics gynecology, Neurosciences neurology, Cardiovascular system cardiology, Infectious diseases, Radiology nuclear medicine medical imaging, Transplantation, Tropical medicine, Allergy, Anesthesiology, Anatomy morphology, General internal medicine, Immunology, Research experimental medicine, Dermatology, Oncology, Surgery.*

### Selection of the studies included in the review

Articles were eligible for inclusion if they were original research articles written in English in peer-reviewed journals reporting an application of GLMM. We excluded articles of statistical methodology development and those that were not entirely involved in clinical medicine (biology, psychology, genetics, sports, dentistry, air pollution, education, economy, family and health politics, computer science, ecology, nutrition, veterinary and nursing).

### Identification of studies

The information from Appendix S1 (Table) was extracted from the selected articles. Data were collected and stored in a database. Then, data were checked to find discrepancies between the two reviewers. Discrepancies were solved by consensus after reviewing again the conflictive articles.

**Figure 1:**
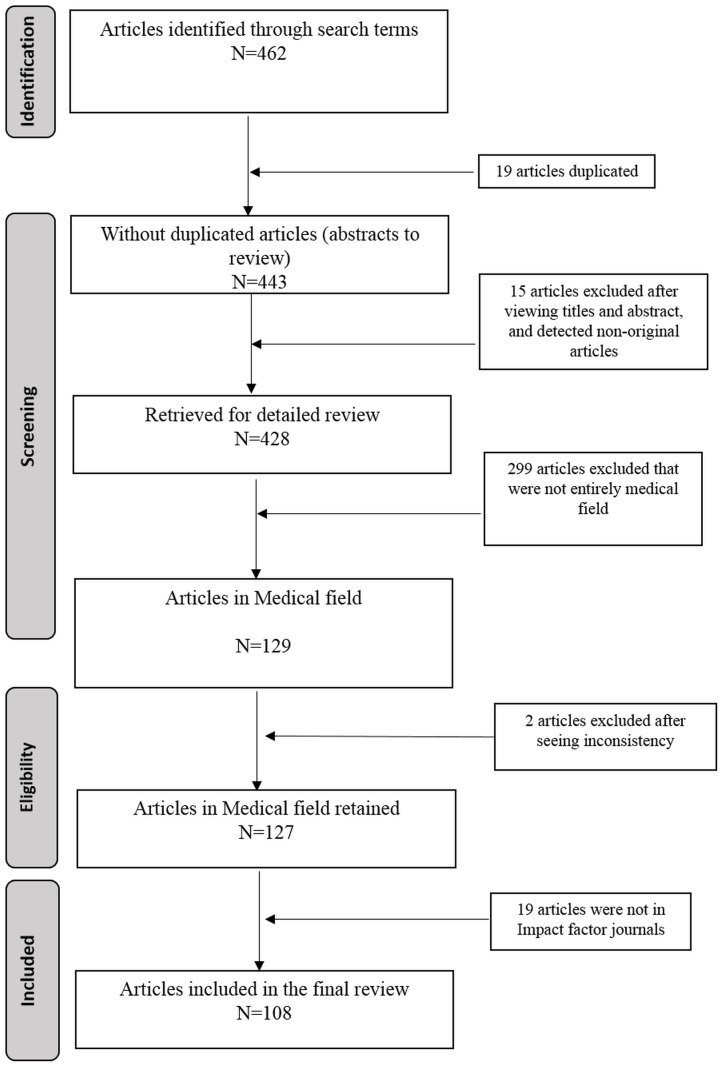
Flow chart of the selection of reviewed articles.

Figure 1 uses the PRISMA flowchart to summarize all stages of the paper selection process [37]. In the first review phase, 462 articles were identified, nineteen of which were duplicates.

After inspection of the abstracts, we excluded the articles that were non-original articles (reviews, short articles or conferences) and those articles that did not have a GLMM as a key word in the abstract or in the title of the article.

In the second review phase, of the 428 articles, only 129 pertained to the aforementioned medical fields. Thus, 299 articles were excluded because they belonged to other fields, such as ecology, computer science, air pollution or statistical methodology. In the third review phase, we obtained full text versions of potentially eligible articles. Two articles were excluded due to inconsistency in the specification of the model applied because in the full text version they were not a GLMM as it was stated in the abstract. We then conducted a detailed review of the 127 articles and we excluded 19 articles because they were not published in an indexed journal included in Journal Citation Reports (JCR).

Finally, 108 articles were included in the final review (Appendix S2). Figure 1 summarizes the numbers of articles identified and the reasons for exclusion at each stage.

### Information collected from the selected articles

Based on Thiele’s and Bolker’s works [22], [38], a list of relevant information and basic characteristics of the study that should be reported in an article with GLMM analysis was suggested (Appendix S1).

### Study characteristics

Regarding the study design, we refer to different aspects of each study, such as hierarchical structure of data and sample size. The hierarchical structure was used to differentiate between the different study designs that are not mutually exclusive, such as longitudinal, repeated measurements, and multilevel studies. Longitudinal data consist of outcome measurements repeatedly taken on each experimental unit over time. Longitudinal analysis is distinct from cross-sectional analysis as it addresses dependency among measurements taken on the same experimental unit [39]. The studies with repeated measurements usually involve only one level of clustering, where the repeated measurements are interchangeable (replicates).

Finally, multilevel studies present various levels of clusters, potentially providing hierarchical structure in each cluster, as seen in longitudinal or repeated measurement studies. We also took note of whether the probability distribution of the variable response was mentioned or easily deducible. Regarding sample size, the number of clusters, individuals or experimental units were collected.

### Inferential issues

This section includes information regarding the GLMM model, as seen in Appendix S1 (Table).

The mixed models are characterized by including fixed and random effects in the linear predictor. Random effects are usually related to the cluster variable. Therefore, it is important to provide information about the cluster variable in the model.

It is also important to report the estimation method of the study and the software applied because they can influence the validity of the GLMM estimates [6], [20], [38]. Furthermore, the software implementations differ considerably in flexibility, computation time and usability [20].

Concerning the computational issues, the macro GLIMMIX from SAS (1992) was the first available software to fit GLMMs using penalized quasilikelihood (PQL) estimation method. The first production version of PROC GLIMMIX for SAS was first released in 2005 and became the standard procedure in version 9.2 in 2008 [40]. Nowadays, there are other available softwares to fit GLMMs. Among them the lme4 package was first implemented for R in 2003 [41]. Moreover, in R software, we can find other packages to fit GLMMs such as glmmML [42], MASS (with the glmmPQL function) [43] or gar (with the repeated function) [44], [45]. Concerning SAS software besides the aforementioned PROC GLIMMIX, the PROC NLMIXED is also able to fit GLMMs [46]. Additionally, it is also possible to use ASReml [47], MLwiN [48] and STATA software (which uses the functions xtmixed and gllamm [22], [28], [49], [50]) [22], [28], [49], [50]. The SPSS (starting with SPSS 19) software now also includes a GLMM obtained in the GENLINMIXED procedure [51], [52].

With respect to statistical inference, the hypotheses concerning fixed and random effects (or their variances) are tested in separated form. Thus, testing the hypotheses for fixed effects is commonly assessed by the Wald score tests. On the other hand, hypotheses concerning random effects variances can be tested using the likelihood ratio test [19] or by comparing the goodness of fit of the models using the Akaike’s Information Criterion (AIC) or the Bayesian Information Criterion (BIC) [19].

### Validation model

Similar to GLMs, validation of GLMMs is commonly based on the inspection of residuals to determine if the model assumptions are fulfilled.

An important point is related to the so-called scale parameter when it is fixed to a specific value because of the probability model assumed. For example, the scale parameter for Poisson and Binomial distribution should be equal to 1. A parameter different from 1 implies that the probability distribution of the responses conditioned to covariates is not correctly specified and the model is not valid. This phenomenon is known as over or underdispersion and causes incorrect standard errors that can produce different clinical conclusions [53]. Thus, it is relevant to evaluate the presence of over- or underdispersion and report the results of this analysis.

Finally, information on the use of a concrete strategy to select the variables in the model and its criterion was obtained. Variable selection strategy usually consist of stepwise selection of variables (forward or backward) [19]. Concerning the criterion, it can be based on entropy as the aforementioned AIC and BIC, or hypotheses testing (likelihood ratio test or Wald test). However, it is possible to find studies with no need of variable selection, for example confirmatory analysis where a particular hypothesized model is fit. This hypothesized model may be based on theory and/or previous analytic research [54], [55]. In this latter case, the selection variable strategy was considered appropriately reported.

## Results

**Figure 2:**
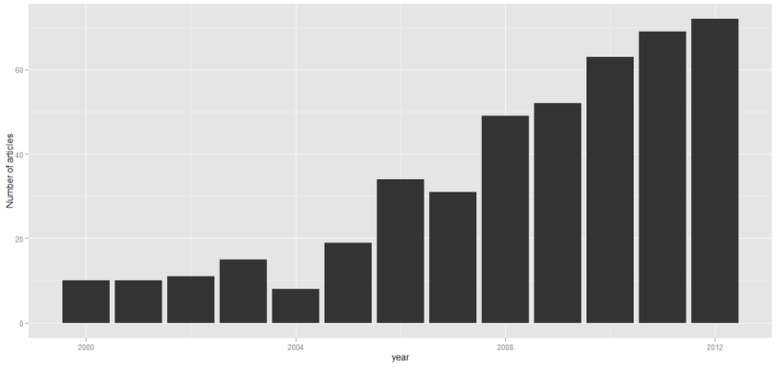
Number of reviewed articles by year of publication.

The evolution of the use of GLMMs in medical journals of the 443 articles selected in the first phase is described in Figure 2. The remaining results (Tables 1, 2, 3 and Appendix S3 and S4) make reference to the 108 articles included in the final in-depth review. Of these, 92 (85.2%) were defined as GLMMs, 14 (13.0%) as HGLMs, and 2 (1.9%) as MGLMs.

**Table 1:** Characteristics of the study design in the reviewed articles.

	N = 108
**Longitudinal study:**	
NO	40 (37.0%)
Unclear	9 (8.30%)
YES	59 (54.6%)
**Repeated measures:**	
NO	34 (31.5%)
Unclear	11 (10.2%)
YES	56 (58.3%)
**Multilevel (nested design):**	
NO	79 (73.1%)
YES	29 (26.9%)
**Type of analysis**	
Exploratory	81 (75.0%)
Confirmatory	27 (25.0%)
**Design**	
Case-control	2 (2.30%)
Case-crossover	1 (1.10%)
Cluster Random Trial	18 (16.7%)
Cohorts	26 (24.1%)
Cross-sectional	31 (28.7%)
NR	8 (7.40%)
Unclear	22 (20.4%)

NR: Not reported.

**Table 2:** Characteristics of inference and estimation methods reported in the review articles.

	N = 108
**Test for fixed effects:**	
NR	103 (95.4%)
t-value	1 (0.90%)
Wald F test	4 (3.7%)
**Test for random effects:**	
LRT	3 (2.80%)
NR	105 (97.2%)
**Variance estimates of random effects:**	
NR	98 (90.7%)
YES	10 (9.30%)
**Statistical software:**	
SAS	57 (52.8%)
R	13 (12.0%)
Stata	12 (11.1%)
WinBugs	2 (1.90%)
S-plus	3 (2.80%)
HLM	6 (5.60%)
Statistical Analysis System	1 (0.90%)
SPSS	2 (1.90%)
SEER*Stat	1 (0.90%)
MLwiN	1 (0.90%)
NR	10 (9.30%)
**Estimation method:**	
Adaptative Quadrature likelihood Approximation	1 (0.90%)
Maximum Likelihood	3 (2.80%)
NR	87 (80.6%)
Penalized Quasi- likelihood	8 (7.50%)
Posterior mean	5 (4.60%)
Restricted Maximum Likelihood	2 (1.90%)
Pseudo likelihood	2 (1.90%)
**Statistical software function or macro:**	
PROC GLIMMIX	24 (22.2%)
glmmPQL	4 (3.70%)
Gllamm	2 (1.90%)
BayesX	2 (1.90%)
Xtmixed	1 (0.90%)
PROC MIXED/NLMIXED	5 (4.70%)
lme4	2 (1.90%)
glmmML	1 (0.90%)
Repeated	1 (0.90%)
NR	66 (61.1%)

NR: No reported; MCMC: Markov chain Monte Carlo.

**Table 3:** Characteristics of the specification, validation and construction of the model for the reviewed articles.

	N = 108
**Variable response distribution:**	
2 distributions: Binomial, Poisson	1 (0.90%)
2 distributions: Binomial, Multinomial	1 (0.90%)
Binomial	64 (59.2%)
Binomial count	1 (0.90%)
Negative Binomial with offset	1 (0.90%)
NR	11 (10.2%)
Poisson	22 (20.4%)
Poisson with offset	2 (1.90%)
Multinomial	2 (1.90%)
Ordinal	1 (0.90%)
Unclear	2 (1.90%)
**Overdispersion evaluation:**	
NR	98 (90.7%)
YES	10 (9.20%)
**Overdispersion measurement:**	
NR	107 (99.1%)
Pearson residuals	1 (0.90%)
**Proposed alternative for overdispersion:**	
GEE	2 (1.90%)
Negative Binomial	2 (1.90%)
NR	100 (92.6%)
Quasi-Poisson	1 (0.90%)
Variogram	1 (0.90%)
Dscale-adjusted	1 (0.90%)
Zero-inflated	1 (0.90%)
**Method of variable selection:**	
Backward	3 (2.80%)
Forward	1 (0.90%)
Forward stepwise	1 (0.90%)
NR	70 (64.8%)
Unnecessary (Confirmatory analysis)	27 (25.0%)
Stepwise	6 (5.60%)
**Method of goodness of fit comparison model:**	
AIC	12 (11.1%)
BIC	3 (2.80%)
DIC	1 (0.90%)
NR	91 (84.3%)
Pseudo R-squared	1 (0.90%)
**GLMM Validation:**	
NR	101 (93.5%)
YES	7 (6.50%)

NR: No reported; MCMC: Markov chain Monte Carlo; GEE: Generalized estimating equation;

DIC: Deviance information criterion; AIC: Akaike information criterion; BIC: Bayesian information criterion; df: freedom degree.

Most of these articles were found in the following journals: *American Journal of Public Health*, which had 7 publications; *PLoS ONE, Cancer Causes & Control*, *BMC Public Health, Annals of Surgery,* and *Headache,* which had 3 publications each. Twenty-two articles pertained to environmental and occupational public health area, 10 articles pertained to clinical neurology, 8 to oncology, and 7 to infectious diseases and pediatrics (Appendix S3).

Forty-five articles (41.7%) were written by an author who was part of a biometric or statistical department and some co-authors (53.3%) were affiliated with a public health department.

Of the 108 selected articles, 59 (54.6%) declared to be longitudinal studies, whereas 56 (58.3%) and 29 (26.9%) were defined as repeated measurements and multilevel design, respectively (Table 1). It is important to note that over 8% of the articles were unclear when reporting the cluster design. Twenty-seven articles (25%) involved confirmatory analysis whereas 81 (75%) were declared as exploratory analysis. Ninety-five of the articles stated their sample size, which ranged from 20–785,385 with a median of 2,201 (Q1 = 408; Q3 = 25000). One random effect in the *intercept* was used in 61 articles, and two or more random effects were used in 36 articles. Of these, 61.1% of the articles had a random effect that pertained to a multilevel model. The size of the random effect or *cluster*, as the number of levels of random effects or the number of clusters, was clearly described in only 33 articles, which ranged from 9–16,230 clusters with a median of 167 (Q1 = 55; Q3 = 1187). The cluster was principally the individual (subject, patient, participant, etc) (n = 46), hospital (n = 15), center (n = 10), geographical area (n = 9) and family (n = 3).

The type of study design was described as cross-sectional (n = 31), cohort (n = 26), clinical trial (n = 18), case-control (n = 2) and *cross-over* (n = 1). Eight articles did not mention study design and 18 articles only described the characteristics of the study design (i.e. experimental, prospective, multicenter, etc) without specifying which study design was used (Table 1).

The response variable (‘clinical’) of the study differed in each of the reviewed articles, and thus there was no common illness or pathology. Available software can fit different response variables for exponential family, such as Poisson, binomial, Gamma, and Inverse Gaussian, though Poisson and Binomial (or binary) are the most used in medicine. The distribution of the response variable was reported in 88% of the articles, and the most common was binomial (n = 64), Poisson (n = 22), negative binomial (n = 1) and multinomial (n = 2).

Furthermore, the estimation method for each model was reported in only 21 articles (19.4%), and the following estimation methods were used: *maximum likelihood* (n = 3), *penalized quasi-likelihood* (n = 8), pseudo-likelihood (n = 2), restricted maximum likelihood (n = 2), *adaptative quadrature likelihood approximation* (n = 1), and Markov chain Monte Carlo (MCMC; n = 5). It is important to mention that over 90% of the articles did not report the test used for the fixed nor random effects, which implies that the section on statistical methods was insufficiently described (Table 2).

The most used statistical software packages were SAS (n = 57), R (n = 13), Stata (n = 12), and HLM (n = 6). For SAS, the use of macro GLIMMIX was reported in 24 articles and the macro NLMIXED with PROC MIXED to fit the GLMM was used in five articles. For R, different packages were used to fit the GLMM, such as lme4 (n = 2), glmmPQL (n = 4), glmmML(n = 1), BayesX (n = 2) or repeated (n = 1). For Stata, the gllamm (n = 2) and xtmixed functions were also used (n = 1).

Overdispersion for models with counts or binary response which assume a Poisson or Binomial distribution was evaluated in 10 articles. Of these, different approaches were proposed to fit as alternatives (GEE, Negative Binomial, Quasi-Poisson, Zero-Inflated). For the articles that used Poisson or Binomial distribution of probability, 90.7% did not state if under-overdispersion was evaluated, 99.1% did not report the magnitude of the scale parameter, and 92.6% did not suggest alternatives for possible under-overdispersion. Variance estimates of random effects were described in only 10 articles (9.3%). With respect to the fixed effects, the standard error and confidence interval were reported in 20% and 71.3%, respectively, whereas in the variance components, they were reported in 3.7% and 2.8%, respectively. The model validation, the method of covariate selection and the method of goodness of fit were reported in 6.5%, 35.2% and 15.7% of the articles, respectively (Table 3).

## Discussion

The articles selected in this review showed that the number of bibliographical references that use GLMMs in medical journals increased from the year 2000 to 2012.

Our review also indicated that there is room for improvement in quality when basic characteristics about the GLMMs are reported in medical journals.

A predominance of the articles reviewed were in the fields of environmental and occupational public health. Furthermore, for 45 of the articles (41.7%) at least one of the co-authors was associated with a biometrics or statistical department. This result is consistent with the systematic review of Diaz-Ordaz that showed that trials having a statistician as co-author was associated with a increase in the methodological quality of the analyses [56].

In any scientific paper, the validity of the conclusions is linked to the adequacy of the methods used to generate the results. Thus, it is important to adequately describe the statistical methods used in the analysis. Hence, the reader is able to judge whether the methods used are appropriate, and by extension whether the conclusions are correct.

In the case of GLMM’s, as we observed in the results section, the majority of the useful and relevant information about GLMMs that is proposed by Bolker [19] and Thiele [22] was not reported. Therefore, the main consequence is the difficulty to assess the reliability of the results and the validity of the conclusions. For example, the majority of the articles did not mention the estimation method or software that was used. The inferential issues (hypothesis testing, confidence interval estimation) and model validation are closely linked to the estimation method (for instance, bayesian or frequentist). As a consequence, the lack of reporting of the estimation method (or software) used makes it complicated to evaluate the adequacy of the approaches used to inference purposes. Furthermore, the estimation method may have important flaws depending on the situation. For example, PQL yields biased parameter estimates if the standard deviations of the random effects are large, especially with binary data [19].

Additionally, an important deficit regarding the inference of fixed and random effects was observed. Such inference may consist of : 1) hypothesis testing of a set of parameters; 2) competing models using entropy measures; 3) confidence interval of parameters. Here again the validity of the conclusions drawn from the analysis depends on the appropriateness of the procedures used in the inference. For example, the likelihood ratio test is only applicable to nested models. Another example arises when testing the existence of a random effect. This question could be solved by a common hypothesis testing using a null hypothesis whose variance is zero. However, the null hypothesis is set to the boundary of the parameter domain (variance must be positive). Therefore, it is necessary to modify the probability distribution function under the null hypothesis otherwise the p-value obtained is incorrect [57]. Additionally, as we mentioned above, the inferential procedures must be coherent with the estimation technique used.

Furthermore, the validity and model selection as proposed by Bolker and Thiele [19], [22] were also not reported in most cases. Once again, the results of the inference and the conclusions of the study will be valid when the assumptions made on the model and estimation method are fulfilled. This is the aim of the validation and, thus, it is essential that the researchers report the results of such a validation and how it was made.

Therefore, in our opinion the methodological information reported in articles using GLMMs could be improved.

We also think that standardized guidelines to report GLMM characteristics in medicine could be beneficial, even though they would not imply by themselves a direct improvement on quality of the articles. As stated by Cobo [35] and Moher [58], it is necessary that both authors and reviewers are aware of recommendations to improve the quality of the manuscripts.

### Limitations of the study

One of the limitations of our study could be that the number of identified articles was not high, despite the 13-years review. Nonetheless, the only similar existing review by Thiele [22] in the field of “invasion biology” included only 50 articles. One possible explanation for this number of articles that use GLMMs in health sciences is that medical literature frequently uses models with fixed effects in a hierarchical structure, even though the use of GLMMs is well known in statistical literature [6], [59].

Another possible limitation of our review is the potential bias to disregard articles that use a GLMM but do not specify the term as a topic. However, we could assume that articles that use GLMM as topic are more sensitive to this methodology. Thus, it is expected that if this bias existed, the reporting quality would be even better in those potential articles that applied GLMM and used it as a topic.

There could be also a trend on the estimation methods according to the names given to GLMMs in the articles. Bayesians usually prefer the term hierarchical models instead of mixed effects models whereas frequentists are more likely to use mixed models, which seems to be consistent with our results (Appendix S4).

## Conclusions

During recent years, the use of GLMMs in medical literature has increased to take into account the correlation of data when modeling binary or count data. Our review included articles from indexed medical journals included in JCR that mainly consisted of longitudinal studies in a medical setting.

According to the current recommendations, the quality of reporting has room for improvement regarding the characteristics of the analysis, estimation method, validation and selection of the model.

After analyzing and reviewing the quality of the publications, we believe it is important to consider the use of minimal rules as standardized guidelines when presenting GLMM results in medical journals.

## Supporting Information

Appendix S1 Search strategy protocol.
(DOCX)

Appendix S2 Articles included in our study.
(DOC)

Appendix S3 Journals according to field of knowledge.
(DOC)

Appendix S4 Estimation methods according to the name used (GLMM, HGLM, MGLM).
(DOC)

Checklist S1 PRISMA Checklist.
(DOC)
