# Supplementary material for: Methodological Quality and Reporting of Generalized Linear Mixed Models in Clinical Medicine (2000–2012): A Systematic Review
Source: PLoS One. 2014 Nov 18;9(11):e112653. doi: 10.1371/journal.pone.0112653 (PMC4236119; doi:10.1371/journal.pone.0112653)
Supplement: Appendix S1 — Search strategy protocol. (DOCX) [file pone.0112653.s001.docx]

**Systematic review of GLMM**

**Databases**

Web of Science database was used.

**Search Terms**

**TOPIC**: ("generalized linear mixed models" OR “generalized linear mixed-effects models” OR “generalized linear models with random effects” OR “ hierarchical generalized linear models” OR “multilevel generalized linear model”)

Refinedby: RESEARCH DOMAINS=( SCIENCE TECHNOLOGY ) AND RESEARCH AREAS=( UROLOGY NEPHROLOGY OR PUBLIC ENVIRONMENTAL OCCUPATIONAL HEALTH OR ONCOLOGY OR GENERAL INTERNAL MEDICINE OR MEDICAL INFORMATICS OR HEALTH CARE SCIENCES SERVICES OR INFECTIOUS DISEASES OR SURGERY OR ORTHOPEDICS OR LIFE SCIENCES BIOMEDICINE OTHER TOPICS OR RESPIRATORY SYSTEM OR ENDOCRINOLOGY METABOLISM OR MEDICAL LABORATORY TECHNOLOGY OR HEMATOLOGY OR PEDIATRICS OR GASTROENTEROLOGY HEPATOLOGY OR ANATOMY MORPHOLOGY OR RHEUMATOLOGY OR OBSTETRICS GYNECOLOGY OR TRANSPLANTATION OR RADIOLOGY NUCLEAR MEDICINE MEDICAL IMAGING OR TROPICAL MEDICINE OR PATHOLOGY OR GERIATRICS GERONTOLOGY OR DERMATOLOGY OR CRITICAL CARE MEDICINE OR CARDIOVASCULAR SYSTEM CARDIOLOGY OR ENTOMOLOGY OR INTEGRATIVE COMPLEMENTARY MEDICINE OR NEUROSCIENCES NEUROLOGY OR RESEARCH EXPERIMENTAL MEDICINE OR EMERGENCY MEDICINE OR ALLERGY OR ANESTHESIOLOGY OR IMMUNOLOGY ) AND PUBLICATION YEARS=( 2011 OR 2006 OR 2004 OR 2009 OR 2007 OR 2000 OR 2005 OR 2008 OR 2001 OR 2012 OR 2002 OR 2010 OR 2003 ) AND DOCUMENT TYPES=( ARTICLE ) AND LANGUAGES=( ENGLISH )

**Date**

2000 to 2012

**Language**

English

**Publication type/status**

Published original articles in peer reviewed journals.

**Study selection**

Stage 1: Screening of titles/ abstracts against inclusion criteria.

Stage 2 and 3: Full papers obtained and assessed against inclusion criteria. Papers will be either accepted or rejected due to failure to meet inclusion criteria and the reason will be specified.

**Inclusion Criteria**

- Original articles written in English that were entirely clinical medical field.

**Exclusion criteria**

- Articles of statistical methodology development and those which were not entirely involved in clinical medicine (biology, psychology, genetics, sports, dentistry, air pollution, education, economy, family and health politics, computer science, ecology, nutrition, veterinary and nursing).
- Inconsistency in the specification of the model applied because in the full text version they were not a GLMM.
- Articles were not in Impact factor journals.

**Data Extraction**

**Table: Information collected from the selected articles.**

| **Characteristics of the study:** |
| --- |
| - Study outcome - Study design - Sample size - Number of clusters - Journals - Field - Authors’ affiliation to a biostatistics or biometric department - Type of analysis (confirmatory/exploratory) |
| **Inferential issues:** |
| - Information about the cluster variable - Estimation method - Statistical software - Statistical software function or macro - Test for fixed effects - Test for random effects - Variance estimates of random effects |
| **Model validation:** |
| - Overdispersion (if assessed and reported) - Method of goodness of fit for model comparison (if necessary) - Method of covariate selection |
