# Supplementary material for: Methodological Quality and Reporting of Generalized Linear Mixed Models in Clinical Medicine (2000–2012): A Systematic Review
Source: PLoS One. 2014 Nov 18;9(11):e112653. doi: 10.1371/journal.pone.0112653 (PMC4236119; doi:10.1371/journal.pone.0112653)
Supplement: Appendix S2 — Articles included in our study. (DOC) [file pone.0112653.s002.doc]

Table: Articles included in our study.

| **References included** |
| --- |
| 1. Abellana R, Ascaso C, Carrasco JL, Castell C, Tresserras R: **Geographical variability of the incidence of Type 1 diabetes in subjects younger than 30 years in Catalonia, Spain.** Med Clin 2009, **132**(12):454-458. |
| 2. Ahmed A, Allman RM, Kiefe CI, Person SD, Shaneyfelt TM, Sims RV, Howard G, DeLong JF: **Association of consultation between generalists and cardiologists with quality and outcomes of heart failure care.** Am Heart J 2003, **145**(6):1086-1093. |
| 3. Andreozzi VL, Bailey TC, Nobre FF, Struchiner CJ, Barreto ML, Assis AMO, Santos LMP: **Random-effects models in investigating the effect of vitamin A in childhood diarrhea.** Ann Epidemiol 2006, **16**(4):241-247. |
| 4. Assassi S, Leyva AL, Mayes MD, Sharif R, Nair DK, Fischbach M, Ngan Nguyen, Reveille JD, Gonzalez EB, McNearney TA, GENISOS Study Grp: **Predictors of Fatigue Severity in Early Systemic Sclerosis: A Prospective Longitudinal Study of the GENISOS Cohort.** Plos One 2011, **6**(10):e26061. |
| 5. Assassi S, Sharif R, Lasky RE, McNearney TA, Estrada-Y-Martin RM, Draeger H, Nair DK, Fritzler MJ, Reveille JD, Arnett FC, Mayes MD, GENISOS Study Grp: **Predictors of interstitial lung disease in early systemic sclerosis: a prospective longitudinal study of the GENISOS cohort.** Arthritis Research & Therapy 2010, **12**(5):R166. |
| 6. Baechle C, Haastert B, Holl RW, Beyer P, Grabert M, Giani G, Icks A, DPV Initiative: **Inpatient and Outpatient Health Care Utilization of Children and Adolescents with Type 1 Diabetes before and after Introduction of DRGs.** Experimental and Clinical Endocrinology & Diabetes 2010, **118**(9):644-648. |
| 7. Bennett KE, Hopper JE, Stuart MA, West M, Drolet BS: **Blood-feeding behavior of vesicular stomatitis virus infected Culicoides sonorensis (Diptera : Ceratopogonidae).** J Med Entomol 2008, **45**(5):921-926. |
| 8. Berdahl TA: **Racial/Ethnic and Gender Differences in Individual Workplace Injury Risk Trajectories: 1988-1998.** Am J Public Health 2008, **98**(12):2258-2263. |
| 9. Boudourakis LD, Wang TS, Roman SA, Desai R, Sosa JA: **Evolution of the Surgeon-Volume, Patient-Outcome Relationship.** Ann Surg 2009, **250**(1):159-165. |
| 10. Boyd HA, Flanders WD, Addiss DG, Waller LA: **Residual spatial correlation between geographically referenced observations - A Bayesian hierarchical modeling approach.** Epidemiology 2005, **16**(4):532-541. |
| 11. Boyd HA, Waller LA, Flanders WD, Beach MJ, Sivilus JS, Lovince R, Lammie PJ, Addiss DG: **Community- and individual-level determinants of Wuchereria bancrofti infection in Leogane Commune, Haiti.** Am J Trop Med Hyg 2004, **70**(3):266-272. |
| 12. Bradley CJ, Dahman B, Bear HD: **Insurance and Inpatient Care Differences in Length of Stay and Costs Between Surgically Treated Cancer Patients.** Cancer 2012, **118**(20):5084-5091. |
| 13. Bradley EH, Herrin J, Wang Y, Barton BA, Webster TR, Mattera JA, Roumanis SA, Curtis JP, Nallamothu BK, Magid DJ, McNamara RL, Parkosewich J, Loeb JM, Krumholz HM: **Strategies for reducing the door-to-balloon time in acute myocardial infarction.** N Engl J Med 2006, **355**(22):2308-2320. |
| 14. Bucher BT, Guth RM, Saito JM, Najaf T, Warner BW: **Impact of Hospital Volume on In-Hospital Mortality of Infants Undergoing Repair of Congenital Diaphragmatic Hernia.** Ann Surg 2010, **252**(4):635-641. |
| 15. Caffrey AR, LaPlante KL: **Changing epidemiology of methicillin-resistant Staphylococcus aureus in the Veterans Affairs Healthcare System, 2002-2009.** Infection 2012, **40**(3):291-297. |
| 16. Campitelli MA, Inoue M, Calzavara AJ, Kwong JC, Guttmann A: **Low Rates of Influenza Immunization in Young Children Under Ontario's Universal Influenza Immunization Program.** Pediatrics 2012, **129**(6):E1421-E1430. |
| 17. Cardo MV, Vezzani D, Carbajo AE: **Environmental Predictors of the Occurrence of Ground Water Mosquito Immatures in the Parana Lower Delta, Argentina.** J Med Entomol 2011, **48**(5):991-998. |
| 18. Cherpitel CJ, Ye Y, Bond J, Rehm J, Poznyak V, Macdonald S, Stafstrom M, Hao W: **Multi-level analysis of alcohol-related injury among emergency department patients: a cross-national study.** Addiction 2005, **100**(12):1840-1850. |
| 19. Childs JD, Teyhen DS, Van Wyngaarden JJ, Dougherty BF, Ladislas BJ, Helton GL, Robinson ME, Wu SS, George SZ: **Predictors of web-based follow-up response in the Prevention of Low Back Pain in the Military Trial (POLM).** Bmc Musculoskeletal Disorders 2011, **12**:132. |
| 20. Chung JH, Phibbs CS, Boscardin WJ, Kominski GF, Ortega AN, Gregory KD, Needleman J: **Examining the effect of hospital-level factors on mortality of very low birth weight infants using multilevel modeling.** Journal of Perinatology 2011, **31**(12):770-775. |
| 21. Cleveland MJ, Feinberg ME, Bontempo DE, Greenberg MT: **The role of risk and protective factors in substance use across adolescence.** Journal of Adolescent Health 2008, **43**(2):157-164. |
| 22. Colford JM,Jr., Hilton JF, Wright CC, Arnold BF, Saha S, Wade TJ, Scott J, Eisenberg JNS: **The Sonoma Water Evaluation Trial: A Randomized Drinking Water Intervention Trial to Reduce Gastrointestinal Illness in Older Adults.** Am J Public Health 2009, **99**(11):1988-1995. |
| 23. Comber H, Sharp L, Timmons A, Keane FBV: **Quality of rectal cancer surgery and its relationship to surgeon and hospital caseload: a population-based study.** Colorectal Disease 2012, **14**(10):E692-E700. |
| 24. Cooper HLF, Des Jarlais DC, Ross Z, Tempalski B, Bossak B, Friedman SR: **Spatial Access to Syringe Exchange Programs and Pharmacies Selling Over-the-Counter Syringes as Predictors of Drug Injectors' Use of Sterile Syringes.** Am J Public Health 2011, **101**(6):1118-1125. |
| 25. Cram P, Bayman L, Popescu I, Vaughan-Sarrazin MS, Cai X, Rosenthal GE: **Uncompensated care provided by for-profit, not-for-profit, and government owned hospitals.** Bmc Health Services Research 2010, **10**:90. |
| 26. Cuadros DF, Branscum AJ, Garcia-Ramos G: **No evidence of association between HIV-1 and malaria in populations with low HIV-1 prevalence.** PloS one 2011, **6**(8):e23458-e23458. |
| 27. Daniak CN, Peretz D, Fine JM, Wang Y, Meinke AK, Hale WB: **Factors associated with time to laparoscopic cholecystectomy for acute cholecystitis.** World Journal of Gastroenterology 2008, **14**(7):1084-1090. |
| 28. Debbink MP, Bader MDM: **Racial Residential Segregation and Low Birth Weight in Michigan's Metropolitan Areas.** Am J Public Health 2011, **101**(9):1714-1720. |
| 29. Dodd CC, Renter DG, Shi X, Alam MJ, Nagaraja TG, Sanderson MW: **Prevalence and Persistence of Salmonella in Cohorts of Feedlot Cattle.** Foodborne Pathogens and Disease 2011, **8**(7):781-789. |
| 30. Dumpa V, Katz K, Northrup V, Bhandari V: **SNIPPV vs NIPPV: does synchronization matter?** Journal of Perinatology 2012, **32**(6):438-442. |
| 31. Egede LE, Gebregziabher M, Lynch CP, Gilbert GE, Echols C: **Longitudinal ethnic differences in multiple cardiovascular risk factor control in a cohort of US adults with diabetes.** Diabetes Res Clin Pract 2011, **94**(3):385-394. |
| 32. Fabio A, Tu L, Loeber R, Cohen J: **Neighborhood Socioeconomic Disadvantage and the Shape of the Age-Crime Curve.** Am J Public Health 2011, **101**:S325-S332. |
| 33. Fernandez de Larrea-Baz N, Alvarez-Martin E, Morant-Ginestar C, Genova-Maleras R, Gil A, Perez-Gomez B, Lopez-Abente G: **Burden of disease due to cancer in Spain.** BMC Public Health 2009, **9**:42-42. |
| 34. Figueiras A, Carracedo-Martinez E, Saez M, Taracido M: **Analysis of case-crossover designs using longitudinal approaches - A simulotion study.** Epidemiology 2005, **16**(2):239-246. |
| 35. Figueiras A, Herdeiro MT, Polonia J, Jesus Gestal-Otero J: **An educational intervention to improve physician reporting of adverse drug reactions - A cluster-randomized controlled trial.** Jama-Journal of the American Medical Association 2006, **296**(9):1086-1093. |
| 36. Filion KB, Steffen LM, Duval S, Jacobs DR,Jr., Blackburn H, Luepker RV: **Trends in Smoking Among Adults From 1980 to 2009: The Minnesota Heart Survey.** Am J Public Health 2012, **102**(4):705-713. |
| 37. Finkelstein JA, Stille C, Nordin J, Davis R, Raebel MA, Roblin D, Go AS, Smith D, Johnson CC, Kleinman K, Chan KA, Platt R: **Reduction in antibiotic use among US children, 1996-2000.** Pediatrics 2003, **112**(3):620-627. |
| 38. Flexeder C, Thiering E, Brueske I, Koletzko S, Bauer C-, Wichmann H-, Mansmann U, von Berg A, Berdel D, Kraemer U, Schaaf B, Lehmann I, Herbarth O, Heinrich J, GINIplus, LISAplus Study Grp: **Growth velocity during infancy and onset of asthma in school-aged children.** Allergy 2012, **67**(2):257-264. |
| 39. Foraker RE, Rose KM, Kucharska-Newton AM, Ni H, Suchindran CM, Whitsel EA: **Variation in Rates of Fatal Coronary Heart Disease by Neighborhood Socioeconomic Status: The Atherosclerosis Risk in Communities Surveillance (1992-2002).** Ann Epidemiol 2011, **21**(8):580-588. |
| 40. Forte ML, Virnig BA, Eberly LE, Swiontkowski MF, Feldman R, Bhandari M, Kane RL: **Provider Factors Associated with Intramedullary Nail Use for Intertrochanteric Hip Fractures.** Journal of Bone and Joint Surgery-American Volume 2010, **92A**(5):1105-1114. |
| 41. Froeschke G, Sommer S: **Insights into the complex associations between MHC class II DRB polymorphism and multiple gastrointestinal parasite infestations in the striped mouse.** PloS one 2012, **7**(2):e31820-e31820. |
| 42. Goodman ER, Platt R, Bass R, Onderdonk AB, Yokoe DS, Huang SS: **Impact of an environmental cleaning intervention on the presence of methicillin-resistant Staphylococcus aureus and vancomycin-resistant enterococci on surfaces in intensive care unit rooms.** Infection Control and Hospital Epidemiology 2008, **29**(7):593-599. |
| 43. Gumpertz ML, Pickle LW, Miller BA, Bell BS: **Geographic patterns of advanced breast cancer in Los Angeles: Associations with biological and sociodemographic factors (United States).** Cancer Causes & Control 2006, **17**(3):325-339. |
| 44. Hall CB, Lipton RB, Tennen H, Haut SR: **Early follow-up data from seizure diaries can be used to predict subsequent seizures in same cohort by borrowing strength across participants.** Epilepsy & Behavior 2009, **14**(3):472-475. |
| 45. Hollingsworth JM, Krein SL, Dunn RL, Wolf JS,Jr., Hollenbeck BK: **Understanding variation in the adoption of a new technology in surgery.** Med Care 2008, **46**(4):366-371. |
| 46. Holmboe ES, Wang Y, Meehan TP, Tate JP, Ho S, Starkey KS, Lipner RS: **Association between maintenance of certification examination scores and quality of care for medicare beneficiaries.** Arch Intern Med 2008, **168**(13):1396-1403. |
| 47. Holmboe ES, Wang Y, Tate JP, Meehan TP: **The effects of patient volume on the quality of diabetic care for Medicare beneficiaries.** Med Care 2006, **44**(12):1073-7. |
| 48. Hsia RY, Kanzaria HK, Srebotnjak T, Maselli J, McCulloch C, Auerbach AD: **Is Emergency Department Closure Resulting in Increased Distance to the Nearest Emergency Department Associated With Increased Inpatient Mortality?** Ann Emerg Med 2012, **60**(6):707-715. |
| 49. Hunter S, Love-Jackson K, Abdulla R, Zhu W, Lee J, Wells KJ, Roetzheim R: **Sun Protection at Elementary Schools: A Cluster Randomized Trial.** J Natl Cancer Inst 2010, **102**(7):484-492. |
| 50. Husted JA, Tom BD, Farewell VT, Schentag CT, Gladman DD: **A longitudinal study of the effect of disease activity and clinical damage on physical function over the course of psoriatic arthritis - Does the effect change over time?** Arthritis Rheum 2007, **56**(3):840-849. |
| 51. Janjua NZ, Skowronski DM, Hottes TS, Osei W, Adams E, Petric M, Lem M, Tang P, De Serres G, Patrick DM, Bowering D: **Transmission dynamics and risk factors for pandemic H1N1-related illness: outbreak investigation in a rural community of British Columbia, Canada.** Influenza and Other Respiratory Viruses 2012, **6**(3):e54-e62. |
| 52. Janjua NZ, Skowronski DM, Hottes TS, Osei W, Adams E, Petric M, Sabaiduc S, Chan T, Mak A, Lem M, Tang P, Patrick DM, De Serres G, Bowering D: **Seasonal Influenza Vaccine and Increased Risk of Pandemic A/H1N1-Related Illness: First Detection of the Association in British Columbia, Canada.** Clinical Infectious Diseases 2010, **51**(9):1017-1027. |
| 53. Jia H, Feng H, Wang X, Wu SS, Chumbler N: **A longitudinal study of health service utilization for diabetes patients in a care coordination home-telehealth programme.** J Telemed Telecare 2011, **17**(3):123-126. |
| 54. Johnson DS, Hoeting JA: **Bayesian multimodel inference for geostatistical regression models.** PloS one 2011, **6**(11):e25677-e25677. |
| 55. Kelley ME, Haas GL, van Kammen DP: **Longitudinal progression of negative symptoms in schizophrenia: A new look at an old problem.** Schizophr Res 2008, **105**(1-3):188-196. |
| 56. Kleinman K, Lazarus R, Platt R: **A generalized linear mixed models approach for detecting incident clusters of disease in small areas, with an application to biological terrorism.** Am J Epidemiol 2004, **159**(3):217-224. |
| 57. Kleinschmidt I, Sharp BL, Clarke GPY, Curtis B, Fraser C: **Use of generalized linear mixed models in the spatial analysis of small-area malaria incidence rates in KwaZulu Natal, South Africa.** Am J Epidemiol 2001, **153**(12):1213-1221. |
| 58. Konety SH, Rosenthal GE, Vaughan-Sarrazin MS: **Surgical volume and outcomes of off-pump coronary artery bypass graft surgery: Does it matter?** J Thorac Cardiovasc Surg 2009, **137**(5):1116-U98. |
| 59. Kravitz RL, Epstein RM, Feldman MD, Franz CE, Azari R, Wilkes MS, Hinton L, Franks P: **Influence of patients' requests for direct-to-consumer advertised antidepressants - A randomized controlled trial.** Jama-Journal of the American Medical Association 2005, **293**(16):1995-2002. |
| 60. Lambertz CK, Johnson CJ, Montgomery PG, Maxwell JR: **Premedication to reduce discomfort during screening mammography.** Radiology 2008, **248**(3):765-772. |
| 61. Lau DT, Mercaldo ND, Harris AT, Trittschuh E, Shega J, Weintraub S: **Polypharmacy and Potentially Inappropriate Medication Use Among Community-dwelling Elders With Dementia.** Alzheimer Disease & Associated Disorders 2010, **24**(1):56-63. |
| 62. Lederer DJ, Kawut SM, Wickersham N, Winterbottom C, Bhorade S, Palmer SM, Lee J, Diamond JM, Wille KM, Weinacker A, Lama VN, Crespo M, Orens JB, Sonett JR, Arcasoy SM, Ware LB, Christie JD, Lung Transplant Outcomes Grp: **Obesity and Primary Graft Dysfunction after Lung Transplantation The Lung Transplant Outcomes Group Obesity Study.** American Journal of Respiratory and Critical Care Medicine 2011, **184**(9):1055-1061. |
| 63. Lewis EC, Mayer JA, Slymen D: **Postal workers' occupational and leisure-time sun safety behaviors (United States).** Cancer Causes & Control 2006, **17**(2):181-186. |
| 64. Lichtman JH, Leifheit-Limson EC, Jones SB, Wang Y, Goldstein LB: **30-Day Risk-Standardized Mortality and Readmission Rates After Ischemic Stroke in Critical Access Hospitals.** Stroke 2012, **43**(10):2741-2747. |
| 65. Lynch BM, Cerin E, Owen N, Aitken JF: **Associations of leisure-time physical activity with quality of life in a large, population-based sample of colorectal cancer survivors.** Cancer Causes & Control 2007, **18**(7):735-742. |
| 66. Lynch BM, Cerin E, Owen N, Hawkes AL, Aitken JF: **Television viewing time of colorectal cancer survivors is associated prospectively with quality of life.** Cancer Causes & Control 2011, **22**(8):1111-1120. |
| 67. Lynch BM, Cerin E, Owen N, Hawkes AL, Aitken JF: **Prospective relationships of physical activity with quality of life among colorectal cancer survivors.** Journal of Clinical Oncology 2008, **26**(27):4480-4487. |
| 68. Mather FJ, Chen VW, Morgan LH, Correa CN, Shaffer JG, Srivastav SK, Rice JC, Blount G, Swalm CM, Wu XC, Scribner RA: **Hierarchical modeling and other spatial analyses in prostate cancer incidence data.** Am J Prev Med 2006, **30**(2):S88-S100. |
| 69. Mayer JA, Woodruff SI, Slymen DJ, Sallis JF, Forster JL, Clapp EJ, Hoerster KD, Pichon LC, Weeks JR, Belch GE, Weinstock MA, Gilmer T: **Adolescents' Use of Indoor Tanning: A Large-Scale Evaluation of Psychosocial, Environmental, and Policy-Level Correlates.** Am J Public Health 2011, **101**(5):930-938. |
| 70. McCall WV, Blocker JN, D'Agostino R,Jr., Kimball J, Boggs N, Lasater B, Rosenquist PB: **Insomnia severity is an indicator of suicidal ideation during a depression clinical trial.** Sleep Med 2010, **11**(9):822-827. |
| 71. McQueen A, Vernon SW, Myers RE, Watts BG, Lee ES, Tilley BC: **Correlates and predictors of colorectal cancer screening among male automotive workers.** Cancer Epidemiology Biomarkers & Prevention 2007, **16**(3):500-509. |
| 72. Molloy SF, Tanner CJ, Kirwan P, Asaolu SO, Smith HV, Nichols RAB, Connelly L, Holland CV: **Sporadic Cryptosporidium infection in Nigerian children: risk factors with species identification.** Epidemiol Infect 2011, **139**(6):946-954. |
| 73. Mueller S, Polley M, Lee B, Kunwar S, Pedain C, Wembacher-Schroeder E, Mittermeyer S, Westphal M, Sampson JH, Vogelbaum MA, Croteau D, Chang SM: **Effect of imaging and catheter characteristics on clinical outcome for patients in the PRECISE study.** J Neurooncol 2011, **101**(2):267-277. |
| 74. Murphy HR, Rayman G, Duffield K, Lewis KS, Kelly S, Johal B, Fowler D, Temple RC: **Changes in the glycemic profiles of women with type 1 and type 2 diabetes during pregnancy.** Diabetes Care 2007, **30**(11):2785-2791. |
| 75. O'Connor PJ, Sperl-Hillen JM, Rush WA, Johnson PE, Amundson GH, Asche SE, Ekstrom HL, Gilmer TP: **Impact of Electronic Health Record Clinical Decision Support on Diabetes Care: A Randomized Trial.** Annals of Family Medicine 2011, **9**(1):12-21. |
| 76. Paintsil E, Ghebremichael M, Romano S, Andiman WA: **Absolute CD4(+) T-lymphocyte count as a surrogate marker of pediatric human immunodeficiency virus disease progression.** Pediatr Infect Dis J 2008, **27**(7):629-635. |
| 77. Partovian C, Gleim SR, Mody PS, Li S, Wang H, Strait KM, Allen LA, Lagu T, Normand ST, Krumholz HM: **Hospital Patterns of Use of Positive Inotropic Agents in Patients With Heart Failure.** J Am Coll Cardiol 2012, **60**(15):1402-1409. |
| 78. Patel MM, Chillrud SN, Correa JC, Hazi Y, Feinberg M, Deepti KC, Prakash S, Ross JM, Levy D, Kinney PL: **Traffic-Related Particulate Matter and Acute Respiratory Symptoms among New York City Area Adolescents.** Environ Health Perspect 2010, **118**(9):1338-1343. |
| 79. Polgreen PM, Bohnett LC, Yang M, Pentella MA, Cavanaugh JE: **A spatial analysis of the spread of mumps: the importance of college students and their spring-break-associated travel.** Epidemiol Infect 2010, **138**(3):434-441. |
| 80. Polgreen PM, Sparks JD, Polgreen LA, Yang M, Harris ML, Pentella MA, Cavanaugh JE: **A statewide outbreak of Cryptosporidium and its association with the distribution of public swimming pools.** Epidemiol Infect 2012, **140**(8):1439-1445. |
| 81. Rao S, Van Donkersgoed J, Bohaychuk V, Besser T, Song X, Wagner B, Hancock D, Renter D, Dargatz D, Morley PS: **Antimicrobial Drug Use and Antimicrobial Resistance in Enteric Bacteria Among Cattle from Alberta Feedlots.** Foodborne Pathogens and Disease 2010, **7**(4):449-457. |
| 82. Regenbogen SE, Gawande AA, Lipsitz SR, Greenberg CC, Jha AK: **Do Differences in Hospital and Surgeon Quality Explain Racial Disparities in Lower-Extremity Vascular Amputations?** Ann Surg 2009, **250**(3):424-431. |
| 83. Roe CM, Xiong C, Miller JP, Cairns NJ, Morris JC: **Interaction of neuritic plaques and education predicts dementia.** Alzheimer Disease & Associated Disorders 2008, **22**(2):188-193. |
| 84. Roe CM, Xiong C, Miller JP, Morris JC: **Education and Alzheimer disease without dementia - Support for the cognitive reserve hypothesis.** Neurology 2007, **68**(3):223-228. |
| 85. Ross JS, Maynard C, Krumholz HM, Sun H, Rumsfeld JS, Normand ST, Wang Y, Fihn SD: **Use of Administrative Claims Models to Assess 30-Day Mortality Among Veterans Health Administration Hospitals.** Med Care 2010, **48**(7):652-658. |
| 86. Rusconi PG, Ludwig DA, Ratnasamy C, Mas R, Harmon WG, Colan SD, Lipshultz SE: **Serial measurements of serum NT-proBNP as markers of left ventricular systolic function and remodeling in children with heart failure.** Am Heart J 2010, **160**(4):776-783. |
| 87. Salhofer-Polanyi S, Frantal S, Brannath W, Seidel S, Woeber-Bingoel C, Woeber C, PAMINA Study Grp: **Prospective Analysis of Factors Related to Migraine Aura - The PAMINA Study.** Headache 2012, **52**(8):1236-1245. |
| 88. Sarnat SE, Raysoni AU, Li W, Holguin F, Johnson BA, Luevano SF, Garcia JH, Sarnat JA: **Air Pollution and Acute Respiratory Response in a Panel of Asthmatic Children along the U.S.-Mexico Border.** Environ Health Perspect 2012, **120**(3):437-444. |
| 89. Schelbert EB, Rosenthal GE, Welke KF, Vaughan-Sarrazin MS: **Treatment variation in older black and white patients undergoing aortic valve replacement.** Circulation 2005, **112**(15):2347-2353. |
| 90. Seetharamaiaha R, West BT, Ignash SJ, Pakarinen MP, Koivusalo A, Rintala RJ, Liu DC, Spencer AU, Skipton K, Geiger JD, Hirschl RB, Coran AG, Teitelbaum DH: **Outcomes in pediatric patients undergoing straight vs J pouch ileoanal anastomosis: a multicenter analysis.** J Pediatr Surg 2009, **44**(7):1410-1417. |
| 91. Sikkema KJ, Wilson PA, Hansen NB, Kochman A, Neyfeld S, Ghebremichael MS, Kershaw T: **Effects of a coping intervention on transmission risk behavior among people living with HIV/AIDS and a history of childhood sexual abuse.** Jaids-Journal of Acquired Immune Deficiency Syndromes 2008, **47**(4):506-513. |
| 92. Svensson J, Johannesen J, Mortensen HB, Nordly S, Danish Childhood Diabet Registry: **Improved metabolic outcome in a Danish diabetic paediatric population aged 0-18 yr: results from a nationwide continuous Registration.** Pediatric Diabetes 2009, **10**(7):461-467. |
| 93. Szyszkowicz M: **Ambient air pollution and daily emergency department visits for headache in Ottawa, Canada.** Headache 2008, **48**(7):1076-1081. |
| 94. Szyszkowicz M: **Air pollution and daily emergency department visits for headache in Montreal, Canada.** Headache 2008, **48**(3):417-423. |
| 95. Thomson BKA, MacRae JM, Barnieh L, Zhang J, MacKay E, Manning MA, Hemmelgarn BR: **Evaluation of an electronic warfarin nomogram for anticoagulation of hemodialysis patients.** Bmc Nephrology 2011, **12**:46. |
| 96. Tran AT, Diep LM, Cooper JG, Claudi T, Straand J, Birkeland K, Ingskog W, Jenum AK: **Quality of care for patients with type 2 diabetes in general practice according to patients' ethnic background: a cross-sectional study from Oslo, Norway.** Bmc Health Services Research 2010, **10**:145. |
| 97. van Baal PH, Engelfriet PM, Hoogenveen RT, Poos MJ, van den Dungen C, Boshuizen HC: **Estimating and comparing incidence and prevalence of chronic diseases by combining GP registry data: the role of uncertainty.** BMC Public Health 2011, **11**:163. |
| 98. Vithiananthan S, Gero D, Zhang JY, Machan JT: **A case-controlled matched-pair cohort study of single-incision and conventional laparoscopic gastric band patients in a single US center with 1-year follow-up.** Surgical Endoscopy and Other Interventional Techniques 2012, **26**(12):3467-3475. |
| 99. Wagner A, Simon C, Oujaa M, Platat C, Schweitzer B, Arveiler D: **Adiponectin is associated with lipid profile and insulin sensitivity in French adolescents.** Diabetes Metab 2008, **34**(5):465-471. |
| 100. Wan ES, Qiu W, Baccarelli A, Carey VJ, Bacherman H, Rennard SI, Agusti A, Anderson WH, Lomas DA, DeMeo DL: **Systemic Steroid Exposure Is Associated with Differential Methylation in Chronic Obstructive Pulmonary Disease.** American Journal of Respiratory and Critical Care Medicine 2012, **186**(12):1248-1255. |
| 101. Williams AL, Khattak AZ, Garza CN, Lasky RE: **The behavioral pain response to heelstick in preterm neonates studied longitudinally: Description, development, determinants, and components.** Early Hum Dev 2009, **85**(6):369-374. |
| 102. Williams ED, Magliano DJ, Zimmet PZ, Kavanagh AM, Stevenson CE, Oldenburg BF, Shaw JE: **Area-Level Socioeconomic Status and Incidence of Abnormal Glucose Metabolism The Australian Diabetes, Obesity and Lifestyle (AusDiab) study.** Diabetes Care 2012, **35**(7):1455-1461. |
| 103. Wright KC, Ravoori MK, Dixon KA, Han L, Singh SP, Liu P, Gupta S, Johnson VE, Kan Z, Kundra V: **Perfusion CT Assessment of Tissue Hemodynamics Following Hepatic Arterial Infusion of Increasing Doses of Angiotensin II in a Rabbit Liver Tumor Model.** Radiology 2011, **260**(3):718-726. |
| 104. Yih WK, Lieu TA, Rego VH, O'Brien MA, Shay DK, Yokoe DS, Platt R: **Attitudes of healthcare workers in US hospitals regarding smallpox vaccination.** BMC Public Health 2003, **3**:20. |
| 105. Zafar AM, Harris TJ, Murphy TP, Machan JT: **Patients' Perspective about Risks and Benefits of Treatment for Peripheral Arterial Disease.** Journal of Vascular and Interventional Radiology 2011, **22**(12):1657-1661. |
| 106. Zeltzer LK, Lu Q, Leisenring W, Tsao JCI, Recklitis C, Armstrong G, Mertens AC, Robison LL, Ness KK: **Psychosocial outcomes and health-related quality of life in adult childhood cancer survivors: A report from the Childhood Cancer Survivor Study.** Cancer Epidemiology Biomarkers & Prevention 2008, **17**(2):435-446. |
| 107. Zhang J, Himes JH, Hannan PJ, Arcan C, Smyth M, Rock BH, Story M: **Summer effects on body mass index (BMI) gain and growth patterns of American Indian children from kindergarten to first grade: a prospective study.** BMC Public Health 2011, **11**:951. |
| 108. Zhu CW, Scarmeas N, Torgan R, Albert M, Brandt J, Blacker D, Sano M, Stern Y: **Clinical characteristics and longitudinal changes of informal cost of Alzheimer's disease in the community.** J Am Geriatr Soc 2006, **54**(10):1596-1602. |
