# Supplementary material for: Methodological Quality and Reporting of Generalized Linear Mixed Models in Clinical Medicine (2000–2012): A Systematic Review
Source: PLoS One. 2014 Nov 18;9(11):e112653. doi: 10.1371/journal.pone.0112653 (PMC4236119; doi:10.1371/journal.pone.0112653)
Supplement: Appendix S3 — Journals according to field of knowledge. (DOC) [file pone.0112653.s003.doc]

Table: Journals according to field of knowledge.

| **AREA** | **N** |
| --- | --- |
| PUBLIC, ENVIRONMENTAL & OCCUPATIONAL HEALTH | 22 |
| CLINICAL NEUROLOGY | 10 |
| ONCOLOGY | 8 |
| INFECTIOUS DISEASES | 7 |
| PEDIATRICS | 7 |
| MEDICINE, GENERAL & INTERNAL | 6 |
| CARDIAC & CARDIOVASCULAR SYSTEMS | 5 |
| ENDOCRINOLOGY & METABOLISM | 5 |
| SURGERY | 5 |
| BIOLOGY | 4 |
| HEALTH CARE SCIENCES & SERVICES | 4 |
| RADIOLOGY, NUCLEAR MEDICINE & MEDICAL IMAGING | 3 |
| CRITICAL CARE MEDICINE | 2 |
| ENTOMOLOGY | 2 |
| ENVIRONMENTAL SCIENCES | 2 |
| FOOD SCIENCE & TECHNOLOGY | 2 |
| GASTROENTEROLOGY & HEPATOLOGY | 2 |
| OBSTETRICS & GYNECOLOGY | 2 |
| RHEUMATOLOGY | 2 |
| ALLERGY | 1 |
| EMERGENCY MEDICINE | 1 |
| GERIATRICS & GERONTOLOGY | 1 |
| ORTHOPEDICS | 1 |
| PSYCHIATRY | 1 |
| SUBSTANCE ABUSE | 1 |
| TROPICAL MEDICINE | 1 |
| UROLOGY & NEPHROLOGY | 1 |
| **Total** | **108** |
