# Supplementary material for: Methodological Quality and Reporting of Generalized Linear Mixed Models in Clinical Medicine (2000–2012): A Systematic Review
Source: PLoS One. 2014 Nov 18;9(11):e112653. doi: 10.1371/journal.pone.0112653 (PMC4236119; doi:10.1371/journal.pone.0112653)
Supplement: Appendix S4 — Estimation methods according to the name used (GLMM, HGLM, MGLM). (DOC) [file pone.0112653.s004.doc]

Table: Estimation methods according to the name used (GLMM, HGLM, MGLM)

| **Estimation method** | **GLMM**  **N=92** | **HGLM**  **N=14** | **MLGM**  **N=2** | **Total** |
| --- | --- | --- | --- | --- |
| Adaptative Quadrature likelihood Approximation | 1 (1.1%) | 0 (0.0%) | 0 (0.0%) | 1 (0,9%) |
| Maximum Likelihood | 3 (3.3%) | 0 (0.0%) | 0 (0.0%) | 3 (2.8%) |
| NR | 74 (80.4%) | 11 (78.6%) | 2 (100%) | 87 (80.6%) |
| Penalized Quasi- likelihood | 8 (8.7%) | 0 (0.0%) | 0 (0.0%) | 8 (7.4%) |
| Posterior mean | 3 (3.3%) | 2 (14.3%) | 0 (0.0%) | 5 (4.6%) |
| Pseudo-likelihood | 1 (1.1%) | 1 (7.1%) | 0 (0.0%) | 2 (1.9%) |
| Restricted Maximum Likelihood | 2 (2,2%) | 0 (0.0%) | 0 (0.0%) | 2 (1.9%) |
